# The draft genome sequence of the grove snail *Cepaea nemoralis*

**DOI:** 10.1093/g3journal/jkaa071

**Published:** 2021-01-11

**Authors:** Suzanne V Saenko, Dick S J Groenenberg, Angus Davison, Menno Schilthuizen

**Affiliations:** 1 Evolutionary Ecology, Naturalis Biodiversity Center, Leiden 2333CR, the Netherlands; 2 Animal Sciences, Institute of Biology Leiden, Leiden University, Leiden 2333BE, the Netherlands; 3 School of Life Sciences, University of Nottingham, Nottingham NG7 2RD, UK

**Keywords:** mollusks, shell pigmentation, supergene, *de novo* assembly and annotation, PacBio sequencing

## Abstract

Studies on the shell color and banding polymorphism of the grove snail *Cepaea nemoralis* and the sister taxon *Cepaea hortensis* have provided compelling evidence for the fundamental role of natural selection in promoting and maintaining intraspecific variation. More recently, *Cepaea* has been the focus of citizen science projects on shell color evolution in relation to climate change and urbanization. *C. nemoralis* is particularly useful for studies on the genetics of shell polymorphism and the evolution of “supergenes,” as well as evo-devo studies of shell biomineralization, because it is relatively easily maintained in captivity. However, an absence of genomic resources for *C. nemoralis* has generally hindered detailed genetic and molecular investigations. We therefore generated ∼23× coverage long-read data for the ∼3.5 Gb genome, and produced a draft assembly composed of 28,537 contigs with the N50 length of 333 kb. Genome completeness, estimated by BUSCO using the metazoa dataset, was 91%. Repetitive regions cover over 77% of the genome. A total of 43,519 protein-coding genes were predicted in the assembled genome, and 97.3% of these were functionally annotated from either sequence homology or protein signature searches. This first assembled and annotated genome sequence for a helicoid snail, a large group that includes edible species, agricultural pests, and parasite hosts, will be a core resource for identifying the loci that determine the shell polymorphism, as well as in a wide range of analyses in evolutionary and developmental biology, and snail biology in general.

## Introduction

**Figure 1:**
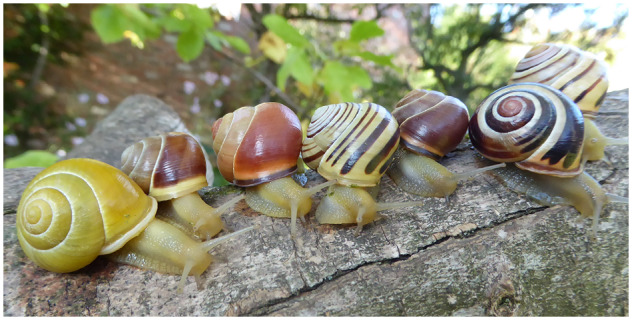
Genetically determined color polymorphism in *C. nemoralis*. Morphs from left to right: (1) yellow unbanded, white lip; (2) brown mid-banded, white lip; (3) pink unbanded, normal lip; (4) yellow five-banded, white lip; (5) brown unbanded, normal lip; (6) pink five-banded, normal lip; (7) yellow three-banded (10305), normal lip. Image credit: Angus Davison.

Studies on the shell color and banding polymorphism of the grove snail *Cepaea nemoralis* (Figure 1), and its sister taxon *Cepaea hortensis*, played a prominent role in demonstrating how selective forces and random processes drive or maintain morphological variation, and contributed to the establishment of the field of ecological genetics (Jones *et al.* 1977; Cook 1998; Ozgo 2008). Alongside the peppered moth, the shell polymorphism of *Cepaea* snails is still *the* classic text book example used to illustrate natural selection and micro‐evolution. Recently, *C. nemoralis* has been the focus of citizen science projects which studied shell color evolution in association to climate change and urbanization (Silvertown *et al.* 2011; Kerstes *et al.* 2019). Being relatively easily maintained and bred in captivity, this snail is also particularly appropriate for evo‐devo studies of shell biomineralization (Mann and Jackson 2014; Jackson and Degnan 2016) and pigmentation (Kerkvliet *et al.* 2017; Affenzeller *et al.* 2020).

Previous work has shown that the shell polymorphism is controlled by a series of nine or more loci, of which five or more are tightly linked in a single “supergene” (Cook 1998; Gonzalez *et al.* 2019). This, combined with the advantages mentioned above, means that *Cepaea* has great potential to provide insights into supergene evolution and the role of genome structure in adaptation. However, progress in understanding the genetic basis of its color pattern formation has been slow, in contrast to other classical systems such as mimicry in *Heliconius* butterflies (Nadeau *et al.* 2016) and industrial melanism in the peppered moth (Van ‘t Hof et al. 2016). Although some advancement toward identifying the supergene has been made recently (Richards *et al.* 2013; Kerkvliet *et al.* 2017), a lack of genomic resources has largely prevented further analyses.

Here, we present a draft assembly and annotation of the *C. nemoralis* genome, the first available genome for helicoid snails (Wade *et al.* 2007) and the second for a terrestrial mollusk, after the giant African snail *Achatina fulica* (Guo *et al.* 2019). Helicoidea is a large group of stylommatophoran land snails that includes not only important models for studies of shell formation and chirality (*e.g.* the genus *Euhadra*, see Davison 2020), but also several edible species (*e.g.* including *Cepaea*, but especially the genera *Helix* and *Cornu*) and agricultural pests. In addition, many Helicoidea are intermediate hosts of various parasites (*e.g.*Gérard *et al.* 2020), and therefore are important subjects in studies of human and animal disease prevention.

Despite great ecological, economical, and medical importance, stylommatophoran land snails have been underrepresented in whole genome sequencing projects (Yang *et al.* 2020), mainly because of their large repetitive genomes (*C*-values between 1.68 and 4.00, see http://www.genomesize.com/). Usually, sequencing coverage above 30× is recommended to overcome this problem (Dominguez Del Angel *et al.* 2018), but this is often financially challenging for individual research groups. Here, we took advantage of recent technological and computational breakthroughs to produce the draft assembly of *C. nemoralis* genome based on lower coverage PacBio sequencing. Even though the assembly presented here is rather fragmented, it should be a key resource for researchers working on diverse aspects of land snail biology, including the identification of genes involved in developmental processes, *e.g.* shell formation and color patterning. Furthermore, it will open up new research avenues for understanding such important biological processes as adaptation to urban environments and climate change, interactions with parasites, and reproduction.

## Materials and methods

### Estimation of genome size by flow cytometry

The haploid chromosome number in *C. nemoralis* is 22 (Page 1978). We performed flow cytometry analysis to estimate the haploid genome size using zebrafish *Danio rerio* as a reference and the “CyStain PI Absolute P” reagent kit (Sysmex Europe, Germany). Briefly, zebrafish tail and snail foot tissues were chopped with a sharp razor blade in 500 µL ice-cold nuclei extraction buffer in a petri dish and incubated for 1 min. Then, the tissues were incubated for 30 minutes in 2.0 mL of staining buffer containing the fluorescent dye propidium iodide (50 µg/mL), RNAse (10 µg/mL), 0.1% dithiothreitol, and 1% polyvinylpyrolidone. The processed sample was passed through a nylon 50 µm filter. The DNA content of stained nuclei was determined using CyFlow-Cube-6 flow cytometer (Sysmex Europe, Germany) as an average of three replicates.

### Sample preparation

A single mid-banded hyalozonate snail with yellow ground color was used for the construction of the reference genome. This individual (C981) is the offspring of cross #13 described in Gonzalez *et al.* (2019), partially inbred, with additional information on and DNA from five generations of the relatives available for future work. High-molecular-weight genomic DNA (HMW-gDNA) was extracted from frozen snail foot tissue using the CTAB (cetyl trimethylammonium bromide) protocol as described in Richards *et al.* (2013) and Gonzalez *et al.* (2019). In brief, slices of snail tissue were incubated at 65°C in extraction solution (3% CTAB, 100 mM Tris‐HCl, pH 7.5, 25 mM EDTA, pH 8, 2 M NaCl) with 0.2 mg/mL proteinase K and 80 μg/mL RNase. Upon lysis, a chloroform extraction was performed, then three volumes of CTAB dilution solution were added (1% CTAB, 50 mm Tris‐HCl, pH 7.5, 10 mM EDTA, pH 8). Samples were mixed until a precipitate appeared, then the supernatant was removed. The pellet was washed twice in 0.4 M NaCl in TE (0.4 M NaCl, 10 mM Tris‐HCl, pH 7.5, 1 mm EDTA, pH 8), redissolved in 1.42 M NaCl in TE (1.42 M NaCl, 10 mM Tris‐HCl, pH 7.5, 1 mM EDTA, pH 8), then precipitated in ethanol, spooled out, washed in 70% ethanol, and air dried. The integrity of extracted HMW-gDNA was evaluated by performing pulsed-field agarose gel electrophoresis, whereas the purity and concentration were measured by spectrophotometry (with Nanodrop 2000, Thermo Fisher Scientific Inc.) and fluorometry (with Qubit 3.0, Thermo Fisher Scientific Inc.), respectively.

### Whole genome sequencing and quality control

We sequenced the genome of *C. nemoralis* using PacBio single-molecule real-time (SMRT) and Illumina platforms. PacBio library preparation and sequencing were performed at Leiden Genome Technology Center (Leiden, the Netherlands). Without additional shearing, 4 µg of HMW-gDNA was converted into a SMRTbell library using “Procedure & Checklist—Preparing >30 kb Libraries Using SMRTbell Express Template Preparation Kit” (Pacific Biosciences). The insert size of the final library was then determined on Fragment Analyzer (Agilent Technologies). To increase the sequencing read length, an additional damage repair was performed on the library. The library was annealed with sequencing primer V4 and binding was done using binding kit version 3. The library was sequenced with 20 h movie-time using Sequel Sequencing kit v3.0 chemistry on 12 PacBio Sequel SMRT cells (PacBio Sequel System, RRID: SCR_017989), generating 7,202,997 subreads, or 80 Gb of sequence data (*i.e.* 23× genome coverage). The polymerase read length N50 (18,196 bp) was only slightly higher than the subread length N50 (16,882 bp), indicating that the majority of data consists of continuous long reads (CLRs). In addition, 17,390 circular consensus sequencing (CCS) reads of >99% accuracy were generated as well.

For Illumina sequencing, HMW-gDNA was sheared with the Covaris M220 (Covaris Inc., Woburn, MA, USA), set to 500-bp fragment size. A paired-end library was prepared using NEBNext Ultra II DNA Library Prep Kit (New England Biolabs) and sequenced on the Illumina NovaSeq 6000 Sequencing System (RRID: SCR_016387). Illumina sequencing was performed at BaseClear B.V. (Leiden, the Netherlands). Initial quality assessment was based on data passing the Illumina Chastity filtering. Subsequently, reads containing PhiX control signal were removed using an in-house filtering protocol. In addition, reads containing (partial) adapters were clipped (up to a minimum read length of 50 bp). The second quality assessment of the remaining reads was done with FASTQC v0.11.5 (Andrews 2014). We obtained ∼400 million of filtered 150 bp paired-end reads, or 120 Gb of sequence data, representing ∼34× coverage of a 3.5 Gb genome.

### Heterozygosity estimation

Illumina paired-end reads were used to estimate heterozygosity of the sequenced individual by *k*-mer analysis. We used Jellyfish v2.3.0 (Jellyfish, RRID: SCR 005491) (Marcais and Kingsford 2011) to count canonical 31-mers from the sequencing data and to produce the *k*-mer count histogram with max coverage threshold set to 1,000,000. The latter was analyzed by GenomeScope (Vurture *et al.* 2017) to estimate the heterozygosity.

### *De novo* genome assembly

The reference genome of *C. nemoralis* was constructed from PacBio CLRs of >5 kb (a total of 4.8 million reads, or 73.7 Gb of sequence data) using three different assembly packages. First, we used Flye v2.4.2 (Flye, RRID: SCR_017016) (Kolmogorov *et al.* 2019) with default parameters for raw PacBio reads to construct a 4.2 Gb genome assembly with 70,762 contigs and a contig N50 length of 105 kb. Then, we used Canu v1.8 (Canu, RRID: SCR_015880) (Koren *et al.* 2017) with parameters adjusted for low coverage and fast overlap (corMhapFilterThreshold = 0.0000000002 corMhapOptions = “–threshold 0.80 –num-hashes 512 –num-min-matches 3 –ordered-sketch-size 1000 –ordered-kmer-size 14 –min-olap-length 2500 –repeat-idf-scale 50” mhapBlockSize = 500 ovlMerDistinct = 0.975 correctedErrorRate = 0.105 corMinCoverage = 0 corMhapSensitivity=high minReadLength = 5000 minOverlapLength = 2500 corOutCoverage = 200) to produce a 4.9 Gb assembly with 66,503 contigs and N50 of 111 kb. Finally, we used the output of Canu trimming stage (4.1 million reads, or 61.5 Gb) and the CCS reads as an input for wtdbg2 v2.4 (WTDBG2, RRID: SCR_017225) (Ruan and Li 2020), which was run with preset parameters for CCS reads and options “–edge-min 2 –rescue-low-cov-edges.” The resultant Wtdbg2 assembly is 3.5 Gb and contained 64,453 contigs with N50 of 132 kb. Assembly statistics assessed using Quast v5.02 (QUAST, RRID: SCR_001228) (Gurevich *et al.* 2013) are shown in Table 1.

**Table 1:** Statistics for different stages of genome assembly

Assembly	Total size, bp	GC (%)	Contig number	No. of contigs > 10 kb	Max. contig length, bp	Contig N50, bp	Contig L50
**Flye**	4,193,822,794	41.38	70,762	54,326	1,622,356	105,488	10,998
**Canu**	4,892,999,477	41.09	66,503	64,936	1,255,215	110,511	12,124
**Wtdbg2**	3,512,271,831	41.16	64,453	52,879	1,739,336	131,562	7,053
**Flye_red**	3,362,274,305	41.36	42,275	37,759	1,622,356	121,492	7,997
**Canu_red**	3,565,230,412	41.16	38,076	37,950	1,255,215	132,263	8,271
**Wtdbg2_red**	3,163,782,079	41.16	44,228	39,951	1,739,336	148,882	5,957
**Final**	3,490,924,950	41.25	28,537	26,580	3,510,238	333,110	3,035

Duplicated contigs were removed from all three assemblies using purge_dups v1.0.0 (Guan *et al.* 2020) with default parameters. These reduced assemblies are characterized by smaller size and contig numbers, and improved contig N50 lengths (Table 1). Next, to produce a more contiguous assembly, we merged the three reduced assemblies in two consecutive steps. First, we aligned Canu_red and Flye_red assemblies using MUMmer v4.0.0 (MUMmer, RRID: SCR_018171) (Kurtz *et al.* 2004) with nucmer parameters “–maxmatch -c 300 -l 100” and delta-filter parameters “-r -q -l 10000,” and merged them using Quickmerge (Chakraborty *et al.* 2016) (parameters “-hco 10 -c 3 -l 130000 -ml 10000”), with Flye_red as a query and Canu_red as reference input. The resultant assembly was used as reference input to merge with the Wtdbg2_red assembly in the second step, with parameters “-hco 10 -c 3 -l 200000 -ml 10000.” Finally, the assembly was polished twice: (1) using the arrow algorithm from PacBo GenomicConsensus package (https://github.com/pacificbiosciences/genomicconsensus/) and PacBio subreads and (2) using Pilon v1.23 (Pilon, RRID: SCR_014731) (Walker *et al.* 2014) with highly accurate Illumina short reads and parameters “–changes –diploid –fix bases –nostrays.”

### Genome assembly quality evaluation

The final assembly was evaluated in three different ways. First, to assess potential contamination in the sequences, we used BlobTools v1.0.1 (Blobtools, RRID: SCR_017618) (Laetsch and Blaxter 2017) with default parameters. The NCBI nonredundant nucleotide database and the UniProt reference proteome database (both downloaded on October 29, 2019) were used for the taxonomy classification step. Hit files were generated by sequence similarity searches against these databases using BLASTn v2.9.0+ (BLASTN, RRID: SCR 001598) (Camacho *et al.* 2009) and Diamond (DIAMOND, RRID: SCR_016071) (Buchfink *et al.* 2015), respectively. Second, we aligned raw Illumina paired-end reads to the assembly with BWA v0.7.16a (BWA, RRID: SCR_010910) (Li and Durbin 2009). Finally, we evaluated gene content completeness using BUSCO v4.0.2 (BUSCO, RRID: SCR_015008) (Simão *et al.* 2015) with the metazoa_odb10 dataset consisting of 954 BUSCOs (Benchmarking Universal Single-Copy Orthologs) from 65 species.

### Repeat element annotation

A species-specific *de novo* library of transposable elements (TEs) and repeats was generated for *C. nemoralis* using RepeatModeler v2.0.1 (RepeatModeler, RRID: SCR_015027) (Smit and Hubley 2008–2015) and its integrated tools RECON (Bao and Eddy 2002), Tandem Repeats Finder v4.09 (Benson 1999), and RepeatScout v1.0.5 (RepeatScout, RRID: SCR_014653) (Price *et al.* 2005). This custom database (Supplemental File S1) was combined with the library of known repeats from other species v24.01 obtained from RepBase (Bao *et al.* 2015). The combined library was used to identify and soft-mask repetitive elements in the *C. nemoralis* genome with RepeatMasker v4.1.0 (RepeatMasker, RRID: SCR 012954) (Smit *et al.* 2013–2015), run with rmblastn v2.9.0+ as search engine.

### Genome annotation

The annotation was performed on the soft-masked assembly to avoid missing (parts of) coding sequences due to overlap with masked areas of the genome. We used the MAKER v2.31.10 pipeline (MAKER, RRID: SCR_005309) (Cantarel *et al.* 2007; Campbell *et al.* 2014) in three consecutive rounds, combining *ab initio* gene predictions with sequence-based evidence. In the first round, the available transcriptome generated from foot and mantle tissues of four *C. nemoralis* snails (147,397 contigs, see Kerkvliet *et al.* 2017), as well as the protein dataset of *A. fulica* snail (23,726 predicted proteins, see Guo *et al.* 2019), were aligned to the genome with BLASTn (BLASTN, RRID: SCR_001598) and BLASTx (BLASTX, RRID: SCR_001653) algorithms from BLAST v2.9.0+ (NCBI BLAST, RRID: SCR_004870), respectively (est2genome and protein2genome options in MAKER configuration file). After further refinement of these alignments with respect to splice sites using Exonerate v2.4.0 (Exonerate, RRID: SCR_016088) (Slater and Birney 2005), MAKER generated gene models and calculated their annotation edit distance (AED) scores in order to assess the quality of gene prediction (*i.e.* the lower AED value the smaller the difference between the predicted protein and the transcript/protein evidence). Out of 308,927 genes models generated in the first round, 89% had an AED <0.5, indicating that the annotation is well-supported by transcript and/or protein evidence.

The second and third rounds of MAKER were performed on the gene models with AED < 0.4 obtained from the first and second runs, respectively. MAKER scripts maker2zff, fathom, forge, and hmm-assembler.pl were used to create snaphmm files (snaphmm option in maker configuration file) to train *ab initio* gene predictor SNAP (SNAP, RRID: SCR_002127) (Korf 2004). Another *ab initio* gene predictor, Augustus v3.3.3 (Augustus, RRID: SCR_008417) (Stanke *et al.* 2006), was self-trained running BUSCO v4.0.2 with the specific parameter (–long); the generated “retraining parameters” file for *C. nemoralis* was included in the second and third rounds of MAKER annotation. The third and final round of MAKER generated 173,620 gene models with AED <0.5. As the annotation was performed on the soft-masked assembly, many of these putative genes could be derived from repetitive sequences, explaining such a high number. Hence, we removed gene models with >50% overlap within a single repeat region as annotated by Repeat Masker (see above). This resulted in the final set of 43,519 predicted protein-coding genes (Supplementary Files S2 and S3) with average AED of 0.27.

We performed functional annotation of predicted proteins using three automated methods. First, we applied Diamond (Buchfink *et al.* 2015) BLASTp searches (–sensitive –max-target-seqs 1 –outfmt 6 qseqid sallseqid pident evalue bitscore -evalue 1e-5) against UniProt reference proteome database (v2019_09, composed of 561,176 Swiss-Prot and 180,179,667 TrEMBL entries) and the NCBI nonredundant protein database (downloaded on 26 May 2020 and composed of 287,467,303 entries). Second, we used KEGG Automatic Annotation Server (KAAS) (Moriya *et al.* 2007) with eukaryotic species set and the bi-directional best-hit method to assign KEGG orthology (Kanehisa *et al.* 2012) to gene models. Finally, we used InterProScan (Jones *et al.* 2014) and Blast2GO (Götz *et al.* 2008) functions in the OmicsBox to examine motifs, domains, and signatures in the protein sequences and to assign gene ontology (GO) terms to the gene models.

### Data availability

This *C. nemoralis* whole genome sequencing project has been submitted to NCBI with BioProject accession number PRJNA646049. Sequencing reads from Illumina and PacBio platforms have been deposited at NCBI Sequence Read Archive (SRA) under the accession numbers SRX8724912 and SRX8724913, respectively. The assembled genome sequence has been deposited at DDBJ/ENA/GenBank under the accession JACEFZ000000000. The version described in this study is version JACEFZ010000000. Supplementary material is available on figshare (https://doi.org/10.25387/g3.13353083). Supplementary File S1 contains *de novo* library of repeats and TEs generated by RepeatModeler. Supplementary File S2 contains sequences of protein-coding genes predicted in the *C. nemoralis* genome with MAKER. Supplementary File S3 contains MAKER annotation results. Supplementary Table S1 contains GenomeScope results. Supplementary Table S2 contains full output of the BlobTools analysis. Supplementary Table S3 contains BUSCO results. Supplementary Table S4 contains RepeatMasker results. Supplementary Table S5 contains functional annotation of the predicted protein-coding genes. Supplementary Figure S1 describes main characteristics of the predicted protein-coding genes.

## Results and discussion

### Genome size and heterozigosity estimation

**Figure 2:**
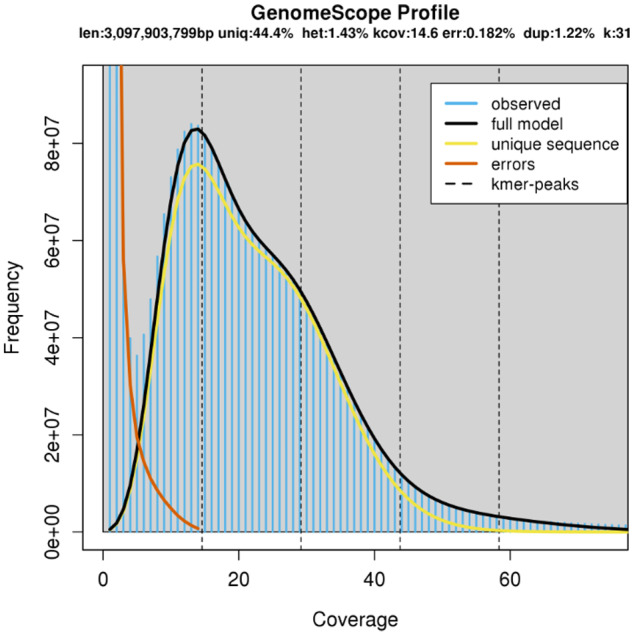
GenomeScope *k*-mer profile plot for the genome of *C. nemoralis* individual C981, based on 31-mers in Illumina reads. The observed *k*-mer frequency distribution is depicted in blue, whereas the GenomeScope fit model is shown as a black line. The unique and putative error *k*-mer distributions are plotted in yellow and red, respectively.

We used flow cytometry to determine that the haploid genome size of *C. nemoralis* is 2.06 times larger than that of the zebrafish (*C*-value ∼1.7, see Vinogradov 1998; Ciudad *et al.* 2002) and is therefore ∼3.5 picogram, or ∼3.42 Gb. When taking the total length of the most recent zebrafish genome assembly of 1.68 Gb (cf. Genome Reference Consortium, https://www.ncbi.nlm.nih.gov/grc/zebrafish/data, last accessed on 13-01-2021) as a reference, the genome size of *C. nemoralis* is calculated at ∼3.46 Gb. This fits within the range of estimated genome sizes for others members of the family Helicidae (*C*-values between 2.88 and 4.00, see http://www.genomesize.com/, last accessed on 13-01-2021). The 31-mer based estimate of genome size provided by GenomeScope (∼3.1 Gb, see Figure 2 and Supplementary Table S1) is smaller than the flow cytometry estimate. Such discrepancy is often found in repeat-rich genomes (*e.g.*Edwards *et al.* 2018), because high-frequency repeats are difficult to model accurately, leading to an underestimation of total repeat length and therefore genome size. The heterozygosity of the individual C981 (Gonzalez *et al.* 2019) genome estimated by GenomeScope (Vurture *et al.* 2017) is ∼1.42%, consistent with the high heterozygosity of other sequenced mollusks (*e.g.*Guo *et al.* 2019; Kenny *et al.* 2020).

### Genome assembly and quality evaluation

We used 4.8 million PacBio long reads, or 73.7 Gb of sequence data, to assemble the genome of *C. nemoralis*. The assembly was polished with PacBio subreads and with highly accurate Illumina short reads. The final genome assembly has total length of 3.5 Gb and is composed of 28,537 contigs with N50 length of 333 kb (Table 1). The mapping rate of Illumina reads agains the final assembly was rather high, with about 99.3% of the reads aligned, and 93.5% properly paired (*i.e.* both reads of the pair mapped to the same contig).

**Figure 3:**
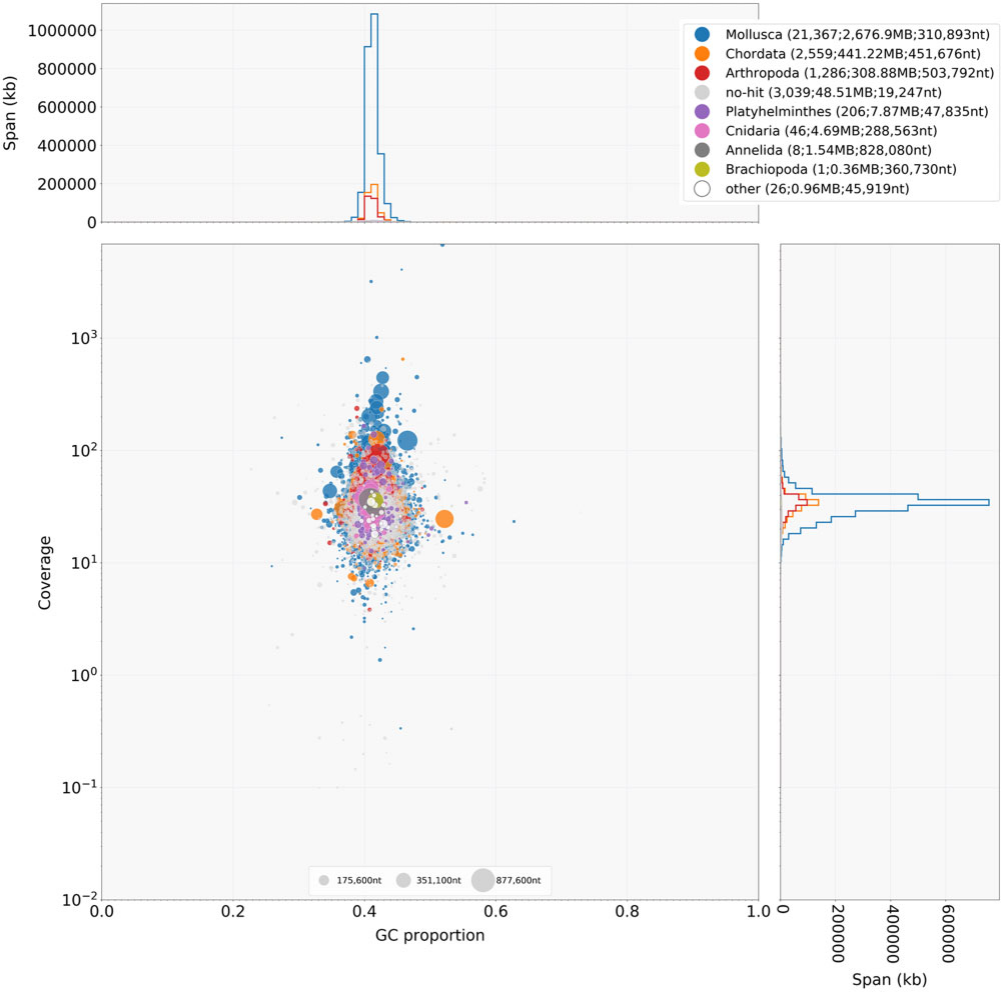
BlobPlot of the *C. nemoralis* genome assembly. Each contig is represented by a circle, colored according to the best match to taxonomic annotation (*e.g.* Mollusca, Chordata, and so on) and distributed according to the proportion GC (*x*-axis) and read coverage (*y*-axis). The upper- and right-hand panels show the distribution of the total span (kb) of contigs for a given coverage (right panel) or GC (upper panel) bin.

Blobtools analyses indicated no substantial contamination with bacterial DNA (Figure 3 and Supplementary Table S2). About 75% of the contigs were assigned to Mollusca, whereas ∼20% were assigned to Chordata and Arthropoda. Closer examination of such cases revealed that the assignment to these two orders is due to a chance blast match with relatively high similarity over a small region of the contig (*i.e.* top hit is to a vertebrate/arthropod species but multiple other hits with a slightly lower bit score are to a mollusk species).

Finally, assembly completeness was assessed with BUSCO v4.0.2 (Simão *et al.* 2015), the tool that looks for Benchmarking Universal Single-Copy Orthologs (BUSCOs) that should be present in a metazoan genome. Out of the 954 metazoan BUSCOs, 832 (87.2%) were identified in the draft assembly of *C. nemoralis* genome as complete (709, or 74.3% as single copy, and 123, or 12.9% as duplicated), 36 (3.8%) as fragmented, and only 86 (9.0%) as missing (Supplementary Table S3). High levels of duplicated genes indicate that, despite haplotig removal, some genomic regions were assembled as separate contigs, most likely due to the high heterozygosity of the genome.

### Genome annotation

**Figure 4:**
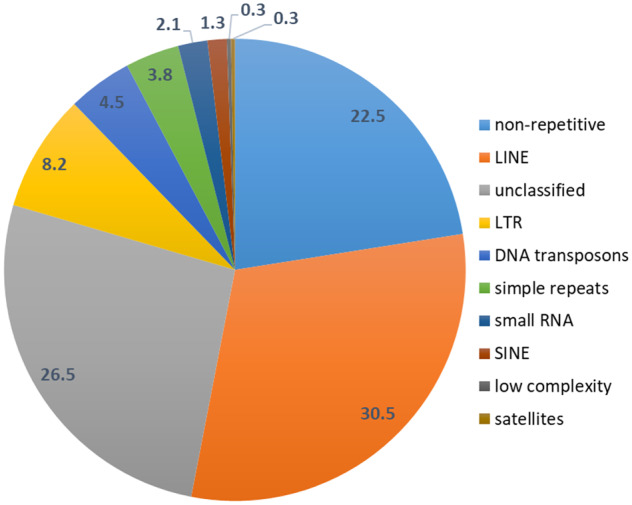
Repetitive content of the assembled *C. nemoralis* genome as identified by RepeatMasker. Numbers indicate percentages of the genome size. NonLTR retrotransposons of the LINE type and LTR retrotransposons, as well as unclassified sequences, dominate the repetitive content.

We estimated the total repeat content of the *C. nemoralis* genome to be around 77% (Figure 4), comparable to the 71% found in *A. fulica* (Guo *et al.* 2019) and expected for such a large genome. Nearly 45% of the genome can be attributed to TEs: nonLTR retrotransposons such as LINEs (long interspersed nuclear elements) and SINEs (short interspersed nuclear elements), LTR (long terminal repeat) retrotransposons, and DNA transposons; ∼6.4% of the repeats were predicted to be small RNAs (*i.e.* transfer RNAs and small nuclear RNAs), satellites, simple and low-complexity repeats (Table 2 and Supplementary Table S4).

**Table 2:** Major types of repeat elements identified in the *C. nemoralis* genome assembly

Repeat class	Repeat element type	No. of elements	Total length, bp	% of genome
**LINE**	RTE/Bov-B	1,922,793	657,283,360	18.83
	R1/LOA/Jockey	378,298	203,267,666	5.82
	L2/CR1/Rex	91,512	44,303,678	1.27
	R2/R4/NeSL	15,845	6,537,107	0.19
**SINE**	Penelope	88,259	27,930,778	0.80
**LTR**	Gypsy/DIRS1	209,476	286,647,606	8.21
**DNA transposon**	Tc1-IS630-Pogo	162,488	58,611,608	1.68
	hobo-Activator	147,068	50,294,093	1.44

We annotated the genome using MAKER v2.31.10 (Cantarel *et al.* 2007; Campbell *et al.* 2014), by supplementing the *ab initio* gene predictions with the *C. nemoralis* transcriptome (Kerkvliet *et al.* 2017) and the protein dataset of the snail *A. fulica* (Guo *et al.* 2019), and two additional rounds of further refinement of gene models with multiple tools integrated into the MAKER pipeline. The final assembly contains 43,519 predicted protein-coding genes (Supplementary Files S2 and S3). Length distribution for genes, exons, and introns is comparable to those of other mollusks (Guo *et al.* 2019) (Table 3 and Supplementary Figure S1). About 93.1% of the predicted genes have multiple exons (4.7 on average), which is slightly lower than in other mollusks (Kenny *et al.* 2020). This could be explained by some degree of fragmentation in the gene models, especially those in small contigs. In addition, 97.3% of the predicted protein-coding genes had a hit to at least one of the databases (Table 4) and were functionally annotated (Supplementary Table S5).

**Table 3:** Characteristics of the annotated genes in the *C. nemoralis* assembly

Feature	Value
Number of protein-coding genes	43,519
Mean gene locus size (bp)	9,296
Mean transcript size (bp)	1,492
Mean exon size (bp)	315
Mean intron size (bp)	2,094
Number of multi-exon genes	40,534
Number of single-exon genes	2,985
Number of distinct exons	205,715
Mean number of distinct exons per gene	4.7

**Table 4:** Summary of functional annotation

Database	Number of hits	%
NCBI nonredundant protein (NR)	37,991	87.3
UniProt (Swiss-Prot and TrEMBL)	37,510	86.2
KEGG orthology	9,342	21.5
InterPro	40,086	92.1
GO terms (InterProScan)	19,288	44.3
GO terms (Blast2GO)	14,866	34.2
Nonredundant hits	42,337	97.3
Unannotated	1,182	2.7

### Conclusions and perspectives

We performed whole-genome assembly of *C. nemoralis* using a combination of PacBio long-read technology and Illumina short-read sequencing. This ∼3.5 Gb draft assembly is composed of 28,537 contigs with the N50 length of 333 kb; repetitive regions cover over 77% of the genome. BUSCO analysis showed that only 9.0% of metazoan orthologs were missing, indicating high genome completeness. More than 43,000 protein-coding genes were identified in the genome, and more than 97.0% of these were functionally annotated from either sequence homology or protein signature searches. To our best knowledge, this is the largest gastropod genome sequenced and assembled to date. Compared to other gastropods (*e.g.* Guo *et al.* 2019; Gomes-dos-Santos *et al.* 2020; Sun *et al.* 2020), the genome of *C. nemoralis* is characterized by a very high content of repetitive sequences.

Despite its large size and the abundance of repeats, the assembly presented here is of high quality, and will be a valuable resource for the land snail research community. In particular, it will facilitate the identification of genes that drive the extraordinary diversity of shell colors and patterns in *C. nemoralis*, and the sister species *C. hortensis*, as well as comparative work in other stylommatophoran snails. In addition, the genome assembly described here will directly enable a wide range of studies on various aspects of terrestrial snail biology, from early development and biomineralization to physiology, behavior, and population genomics.
